# Association of a data-driven nutrient risk score with incident rheumatoid arthritis in UK Biobank adults: a prospective cohort study

**DOI:** 10.3389/fimmu.2026.1831599

**Published:** 2026-05-26

**Authors:** Aimin Wang, Wenfeng Gao, Xinyu Yang, Yanxia Wang, Yonghua Ma, Na Sun, Suzhen Wang, Peizhong Peter Wang, Fuyan Shi

**Affiliations:** 1 School of Public Health, Shandong Second Medical University, Weifang, China; 2 Department of Rheumatology and Immunology, Affiliated Hospital of Shandong Second Medical University, Weifang, China; 3 Division of Community Health and Humanities, Faculty of Medicine, Memorial University of Newfoundland, St. John’s, NL, Canada; 4 Dalla Lana School of Public Health, University of Toronto, Toronto, ON, Canada

**Keywords:** cohort study, genetic susceptibility, nutrient risk score, nutrients, obesity, rheumatoid arthritis

## Abstract

**Background:**

Although specific nutrients have been linked to rheumatoid arthritis (RA), the impact of overall nutrient intake patterns remains unclear. Here, we aimed to explore the association between the nutrient risk score (NRS) and incident RA, as well as the modifying effect of genetic susceptibility and the potential mediating role of obesity.

**Methods:**

This prospective study included 161,725 participants from the UK Biobank. Nutrients were selected using a LASSO-Cox model. We used Cox proportional hazards models to investigate the association between NRS and the risk of RA. Generalized propensity score (GPS) and inverse probability weighting (IPW) methods were employed to control for potential confounding factors. Stratified analyses were conducted to assess effect heterogeneity across subgroups. Mediation analysis was used to estimate the mediating effect of obesity.

**Results:**

During a median follow-up of 13.4 years, 1,756 participants developed RA. Each unit increase in NRS was associated with a 96% higher risk of RA (HR: 1.96, 95% CI: 1.64-2.33) after adjusting for covariates. The association remained robust in analyses using GPS and IPW weighting. Stratified analyses showed a more pronounced association among individuals reporting short sleep duration (<7 hours) and low levels of physical activity. Furthermore, a gradient association between NRS and RA risk was observed across genetic risk strata. Specifically, compared with participants at low genetic risk with the lowest NRS (Q1), those at high genetic risk with the highest NRS (Q4) had nearly a threefold higher risk of incident RA (HR: 3.28, 95% CI: 2.49-4.32). In addition, several obesity indicators showed potential mediating effects, with the proportion mediated ranging from 3.81% to 13.57%.

**Conclusion:**

Higher NRS was associated with an increased risk of incident RA, especially among individuals with high genetic susceptibility. Obesity partially mediated the association, highlighting NRS as a potential tool for RA risk stratification and intervention.

## 1 Introduction

Rheumatoid arthritis (RA) is an autoimmune disease characterized by joint swelling and pain, which can lead to joint dysfunction, disability, and reduced quality of life in severe cases (1, 2). The 2019 Global Burden of Disease (GBD) study reported that approximately 17.6 million individuals worldwide were affected by rheumatoid arthritis, representing a 14.1% increase compared with 1990. It is estimated that by 2050, 31.7 million people globally will be affected, making it a major public health issue that demands urgent attention (3). RA can also lead to reduced life expectancy (4). Therefore, early identification of biomarkers and modifiable factors is crucial for alleviating the burden of RA.

RA is a multifactorial disease influenced by genetic, environmental, and lifestyle factors (5). In recent years, a growing body of evidence has demonstrated that dietary patterns play a critical role in the risk and progression of RA. Different nutrients and their combinations can significantly influence the development of diseases by regulating immune function (6). Various nutrients (e.g., vitamins, fatty acids, and minerals) have been identified as being associated with the incidence and prognosis of RA in numerous observational studies (7–10), Mendelian randomization analyses (11–13), and animal experiments (14). Due to the complexity and heterogeneity of RA and the relatively small or moderate association of a single nutrient with RA risk, integrating multiple nutrients may explain a larger proportion of RA risk. Therefore, a large prospective cohort study is warranted to evaluate the relationship between comprehensive nutrient intake and RA risk.

Genetic susceptibility is a key determinant of the onset of RA and can be quantified using polygenic risk scores (PRS), which facilitate the identification of individuals with a higher genetic risk of RA (15, 16). Although some studies have shown that the intake of individual nutrients may alleviate the harmful effects of high genetic risk (7), it remains unclear whether genetic susceptibility modifies the association between comprehensive multi-nutrient intake and RA risk.

Previous studies have fully demonstrated a close association between dietary habits and obesity (17–19). As an important metabolic phenotype, obesity is considered to potentially mediate the relationship between nutritional exposures and various disease outcomes. For example, a cross-sectional study reported that obesity might partially mediate the relationship between nutritional patterns and hyperuricemia (20). However, it remains unclear whether obesity also mediates the association between nutritional exposure and the risk of RA, and this remains to be clarified.

Therefore, based on the UK Biobank, this study aimed to achieve three main objectives: 1) to construct a nutrient risk score (NRS) and estimate its association with RA risk; 2) to assess the joint association of genetic susceptibility and NRS with RA risk; and 3) to examine the potential mediating role of various obesity-related indicators.

## 2 Methods

### 2.1 Study population

This study used data from the UK Biobank (Project ID 78500), a large prospective cohort that recruited over 500,000 participants aged 37–73 years across the United Kingdom between 2006 and 2010 (21). Participants attended one of 22 assessment centers and underwent comprehensive evaluations, including physical measurements, biochemical blood tests, and genetic analyses. In addition, detailed information on sociodemographic characteristics, lifestyle factors, early-life exposures, and psychosocial variables was obtained through structured questionnaires and personal interviews (22). Further details are available at www.ukbiobank.ac.uk.

Among the initial 502,366 participants, 163,558 completed at least one 24-hour dietary assessment with no implausible values. Subsequently, 1,833 participants with prior RA were further excluded, leaving 161,725 participants included in the study. See **Supplementary Figure 1** for inclusion and exclusion criteria.

### 2.2 Nutrient quantification and processing

Dietary intake was assessed using the Oxford WebQ, a web-based 24-hour dietary questionnaire used in the UK Biobank, which records consumption of 206 foods and 32 beverages during the previous 24 hours (23, 24). Its reliability has been confirmed in multiple validation studies. Participants completed the questionnaire at baseline and up to four additional time points between April 2009 and June 2012 (25). In the present study, the number of dietary assessments per participant ranged from 1 to 5, with most participants completing 1–3 assessments (1: 33.9%, 2: 23.6%, 3: 22.4%, 4: 16.8%, 5: 3.2%; Mean =2.32, SD = 1.19). For those with repeated assessments, the average daily intake of each nutrient was calculated across all available measurements to better reflect habitual dietary intake and reduce within-person variability.

A total of 63 nutrients were assessed. We categorized these nutrients into 8 groups, including 4 carotenoids, 5 fatty acids, 10 macronutrients, 14 minerals, 3 energies, 10 sugars, 13 vitamins, and 4 others (**Supplementary Table 1**). During preprocessing, outliers beyond ±3 standard deviations were removed, after which variables were natural log-transformed (log [x + 1]) and standardized using Z-scores.

### 2.3 Construction of nutrient risk score

Before constructing the NRS, candidate nutrient variables were screened to avoid information redundancy and multicollinearity. Nutrients that were highly correlated or comprehensive indicators that contain more specific components (e.g., total protein which includes both animal and plant protein) were excluded to prevent redundancy. To identify nutrients most strongly associated with RA onset under multi-nutrient exposure, a least absolute shrinkage and selection operator combined with Cox proportional hazards model (LASSO–Cox) was applied. The penalty parameter (λ) was selected using 10-fold cross-validation based on the minimum partial likelihood deviance criterion, yielding an optimal λ of 0.0001160442 (**Supplementary Figures 2**, **3**). Model discrimination was assessed using the concordance index (C-index), with internal validation performed via 10-fold cross-validation (C-index = 0.755), indicating acceptable discriminative performance. To further improve the robustness of variable selection, a stability selection procedure based on resampling was conducted, and only nutrients selected in more than 80% of iterations were retained. A total of 20 nutrients were identified through this combined approach (**Supplementary Table 2**), which were then entered into a multivariable Cox regression model with stepwise selection to determine the optimal model.

The final model retained 14 nutrients, which were used to construct the NRS as a weighted sum of standardized nutrient intakes: NRS=(−0.11×alcohol)+(−0.07×vegetable protein)+(−0.08×β−carotene)+(−0.11×selenium)+(0.10×pantothenic acid)+(−0.08×β−cryptoxanthin)+(−0.05×maltose)+(0.11×cholesterol)+(−0.12×monounsaturated fatty acids)+(0.09×sodium)+(0.07×haem iron)+(−0.13×niacin equivalent)+(0.08×potassium)+(−0.06×biotin)**Supplementary Table 3**). The NRS is intended to reflect the overall nutrient exposure profile associated with RA risk, capturing the combined contribution of multiple nutrients. All nutrient variables included in the NRS were standardized (Z-scores), so that the score reflects relative differences in nutrient intake on a comparable scale. Accordingly, a one-unist increase in the NRS corresponds to a shift in the combined contribution of multiple nutrients on this standardized scale. The coefficients represent the regression coefficients from the multivariable Cox model and correspond to the log hazard ratio for incident RA associated with a one standard deviation increase in each nutrient. Thus, nutrients with positive coefficients were interpreted as risk-enhancing factors, whereas those with negative coefficients were considered protective factors. Additionally, participants were categorized into four groups based on NRS quartiles: Q1 (<−0.184), Q2 (−0.184 to −0.008), Q3 (−0.008 to 0.176), and Q4 (≥0.176).

### 2.4 Ascertainment of RA

The primary endpoint was incident RA, defined according to the International Classification of Diseases, 10th Revision (ICD-10) codes M05 (seropositive RA) and M06 (other RA) (4). Diagnosis information was obtained predominantly from primary care records, hospital admissions, and self-reported health conditions. The earliest recorded diagnosis date of RA was defined as the event date in this study. The follow-up period for this study ended on 1 May 2023. The follow-up time was defined as the time from the recruitment date to RA diagnosis, death or the end of the current follow-up.

### 2.5 Assessment of genetic risk

Genetic risk for RA was assessed using the standard polygenic risk score (PRS) released by the UK Biobank, derived from external GWAS meta-analysis summary statistics via a Bayesian approach (26). We further divided participants into three groups according to the PRS tertiles: low (tertile 1, < −0.345), medium (tertile 2, −0.345 to 0.511), and high (tertile 3, ≥ 0.511).

### 2.6 Assessment of obesity

Obesity can be quantified using multiple indices reflecting different dimensions, including body mass index (BMI), waist circumference (WC), waist-to-height ratio (WHtR), body roundness index (BRI) and a body shape index (ABSI) for body shape; visceral adiposity index (VAI) and lipid accumulation product (LAP) for visceral fat accumulation; triglyceride-glucose index (TyG) and its derivatives (TyG-BMI, TyG-WC, TyG-WHtR) for metabolic function; and body fat percentage (BF%) for overall adiposity (27). Detailed calculation methods are provided in **Supplementary Table 4**.

### 2.7 Covariates

Based on prior literature and the directed acyclic graph (DAG) (**Supplementary Figure 4**), we selected a set of potential confounders associated with both nutrient intake and RA risk (1, 28). These covariates included age, sex, ethnicity, education, Townsend deprivation index (TDI), smoking status, physical activity (meeting 2017 UK guidelines: ≥150 min moderate/walking or 75 min vigorous per week), sleep duration, hypertension, diabetes and hyperlipidemia.

### 2.8 Statistical analysis

Baseline characteristics were summarized using descriptive statistics. Continuous variables were presented as medians with interquartile ranges (IQRs) and compared using the Mann-Whitney U test, while categorical variables were expressed as frequencies and percentages and compared using Pearson’s chi-square test. Apart from the relatively higher missing rates of physical activity (17.63%), TyG-related indices (13.95%–14.16%) and VAI (14.12%), the remaining variables exhibited comparatively low levels of missingness (less than 5%). Details are presented in **Supplementary Table 5**. Variables with a missing rate of less than 20% were imputed using multiple imputation by chained equations (MICE) implemented in the ‘mice’ package in R. 

Cox proportional hazards models were used to examine the associations between individual nutrient intake, NRS, and RA risk. The models were adjusted for age, sex, ethnicity, education, TDI, smoking status, physical activity, sleep duration, hypertension, diabetes, and hyperlipidemia. The results were presented in the form of hazard ratios (HR)s and 95% confidence intervals (CI)s. The proportional hazards assumption for each Cox regression model was verified using Schoenfeld residuals and no violations were detected (*P*>0.05). To explore potential nonlinear effects of NRS on RA risk, restricted cubic spline (RCS) models with five knots (5th, 25th, 50th, 75th, and 95th percentiles) were applied.

To assess the potential causal relationship between NRS and RA risk, we used causal inference methods based on the generalized propensity score (GPS) in addition to Cox proportional hazards regression. GPS, which extends the propensity score approach to continuous exposures, represents the conditional probability of an individual having their observed NRS level given a set of covariates (29–31). First, GPS was included as an adjustment variable in Cox models to account for systematic differences in confounders across NRS levels. Next, inverse probability weighting (IPW) using GPS was applied to create a pseudo-population in which covariates were balanced across NRS levels, and a weighted Cox model was used to estimate the marginal effect of NRS on RA risk (31, 32). Covariate balance after weighting was assessed using standardized mean differences to ensure adequate comparability across exposure levels. To avoid instability from extreme weights, IPW values were truncated at the 1st and 99th percentiles, and the model was refitted. The consistency of results across these approaches supports the robustness of findings.

Subgroup analyses were conducted by stratifying participants according to age, sex, ethnicity, education, smoking status, physical activity, sleep duration, hypertension, diabetes, and hyperlipidemia. Sensitivity analyses were conducted to test the stability of the findings, including: (1) excluding participants with missing data; (2) excluding those diagnosed with RA within the first two years to reduce potential reverse causation; and (3) using a Fine–Gray competing risk model to account for the influence of death before RA onset.

We further investigated the interaction between the nutrient risk score (NRS) and genetic factors in relation to RA incidence. Multiplicative interaction was examined by incorporating a product term (NRS × PRS) into the Cox proportional hazards model, to evaluated whether the relative effect of NRS on RA risk differed across different levels of genetic susceptibility. Additive interaction was assessed using the relative excess risk due to interaction (RERI), the attributable proportion due to interaction (AP), and the synergy index (SI), which quantify departures from additivity on an absolute risk scale. Evidence of additive interaction was considered present if the 95% CIs for RERI or AP did not include 0, or if the CI for SI did not include 1 (33). All calculations for RERI, AP, and SI were conducted using the ‘interactionR’ package in R.

Mediation analysis was conducted with the ‘CMAverse’ package in R to evaluate whether obesity acted as a mediator in the association between NRS and RA risk. The mediation effect was quantified as the proportion of the total effect mediated via the indirect pathway, with 95% CIs and *P*-values estimated using the bootstrap method.

All statistical analyses were carried out using R (version 4.4.3). Statistical significance was set at a two-tailed threshold of P< 0.05 throughout the study.

## 3 Results

### 3.1 Baseline characteristics

A total of 161,725 participants (94,406 female and 67,319 male) were included in this study, with a median age of 57 years (IQR: 50-63). During a median follow-up of 13.4 years, 1,756 incident cases of RA were documented. Baseline characteristics are presented in **Table 1**. Compared with participants who did not develop RA, those with RA were generally older, predominantly female, and had lower educational attainment. They were also more likely to be former or current smokers and to engage in less physical activity. In addition, these individuals tended to have shorter sleep duration, higher NRS and TDI values, and had a greater prevalence of hyperlipidemia, hypertension, and diabetes. Among the 10 obesity-related indicators, all except ABSI were higher in participants who developed RA.

**Table 1:** Characteristics of the study participants categorized by the incidence of RA.

Characteristic	Overall N = 161,725	Non-RA N = 159,969	RA N = 1,756	*P*-value
NRS, Median (IQR)	-0.01 (-0.18 – 0.18)	-0.01 (-0.18 – 0.17)	0.07 (-0.12 – 0.25)	<0.001
Age, Median (IQR)	57.00 (50.00 – 63.00)	57.00 (50.00 – 63.00)	60.00 (54.00 – 64.00)	<0.001
Sex, n (%)				<0.001
Female	94,406 (58.37)	93,174 (58.25)	1,232 (70.16)	
Male	67,319 (41.63)	66,795 (41.75)	524 (29.84)	
Ethnicity, n (%)				0.25
White	155,399 (96.09)	153,721 (96.09)	1,678 (95.56)	
Others	6,326 (3.91)	6,248 (3.91)	78 (4.44)	
Education, n (%)				<0.001
College or University degree	70648 (43.68)	70041 (43.78)	607 (34.57)	
Others	91,077 (56.32)	89,928 (56.22)	1,149 (65.43)	
Smoking status, n (%)				<0.001
Never	93,985 (58.11)	93,142 (58.23)	843 (48.01)	
Former	56,495 (34.93)	55,752 (34.85)	743 (42.31)	
Current	11,245 (6.95)	11,075 (6.92)	170 (9.68)	
Physical activity, n (%)				<0.001
No	29735 (18.39)	29353 (18.35)	382 (21.75)	
Yes	131,990 (81.61)	130,616 (81.65)	1,374 (78.25)	
Sleep duration, Median (IQR)	7.00 (7.00 – 8.00)	7.00 (7.00 – 8.00)	7.00 (6.00 – 8.00)	0.002
TDI, Median (IQR)	-2.38 (-3.76 – -0.08)	-2.38 (-3.77 – -0.08)	-2.24 (-3.67 – 0.18)	0.011
Hyperlipidemia, n (%)				<0.001
No	140180 (86.68)	138765 (86.74)	1415 (80.58)	
Yes	21,545 (13.32)	21,204 (13.26)	341 (19.42)	
Hypertension, n (%)				<0.001
No	123,550 (76.40)	122,375 (76.50)	1,175 (66.91)	
Yes	38,175 (23.60)	37,594 (23.50)	581 (33.09)	
Diabetes, n (%)				<0.001
No	155,217 (95.98)	153,595 (96.02)	1,622 (92.37)	
Yes	6,508 (4.02)	6,374 (3.98)	134 (7.63)	
PRS, n (%)				<0.001
Low	53,909 (33.33)	53,479 (33.43)	430 (24.49)	
Medium	53,908 (33.33)	53,391 (33.38)	517 (29.44)	
High	53,908 (33.33)	53,099 (33.19)	809 (46.07)	
BMI, Median (IQR)	26.09 (23.63 – 29.14)	26.08 (23.63 – 29.11)	27.19 (24.32 – 31.02)	<0.001
WC, Median (IQR)	88.00 (78.00 – 97.00)	88.00 (78.00 – 97.00)	90.00 (80.00 – 100.00)	<0.001
WHtR, Median (IQR)	0.52 (0.47 – 0.57)	0.52 (0.47 – 0.57)	0.54 (0.49 – 0.60)	<0.001
BRI, Median (IQR)	3.70 (2.84 – 4.70)	3.69 (2.84 – 4.70)	4.14 (3.11 – 5.37)	<0.001
ABSI, Median (IQR)	76.18 (72.14 – 80.01)	76.18 (72.13 – 80.00)	76.01 (72.30 – 80.32)	0.29
VAI, Median (IQR)	1.60 (1.01 – 2.53)	1.60 (1.01 – 2.53)	1.79 (1.10 – 2.82)	<0.001
LAP, Median (IQR)	37.88 (20.86 – 65.10)	37.80 (20.83 – 64.98)	44.58 (24.83 – 76.13)	<0.001
TyG, Median (IQR)	8.64 (8.25 – 9.07)	8.64 (8.25 – 9.07)	8.66 (8.29 – 9.09)	0.044
TyG-BMI, Median (IQR)	226.98 (201.30 – 258.06)	226.88 (201.22 – 257.86)	238.71 (208.53 – 274.93)	<0.001
TyG-WC, Median (IQR)	759.85 (669.06 – 855.95)	759.51 (668.95 – 855.54)	782.83 (686.31 – 888.84)	<0.001
TyG-WHtR, Median (IQR)	4.50 (4.01 – 5.02)	4.49 (4.01 – 5.02)	4.71 (4.16 – 5.29)	<0.001
BF%, Median (IQR)	30.90 (25.00 – 37.30)	30.80 (24.90 – 37.20)	34.60 (28.10 – 40.60)	<0.001

NRS, nutrient risk score; TDI, Townsend deprivation index; PRS, polygenic risk score; BMI, body mass index; WC, waist circumference; WHtR, waist-to-height ratio; BRI, body roundness index; ABSI, a body shape index; VAI, visceral adiposity index; LAP, lipid accumulation product; TyG, triglyceride-glucose index; BF%, body fat percentage.

### 3.2 Associations between nutrients and RA risk

**Figure 1:**
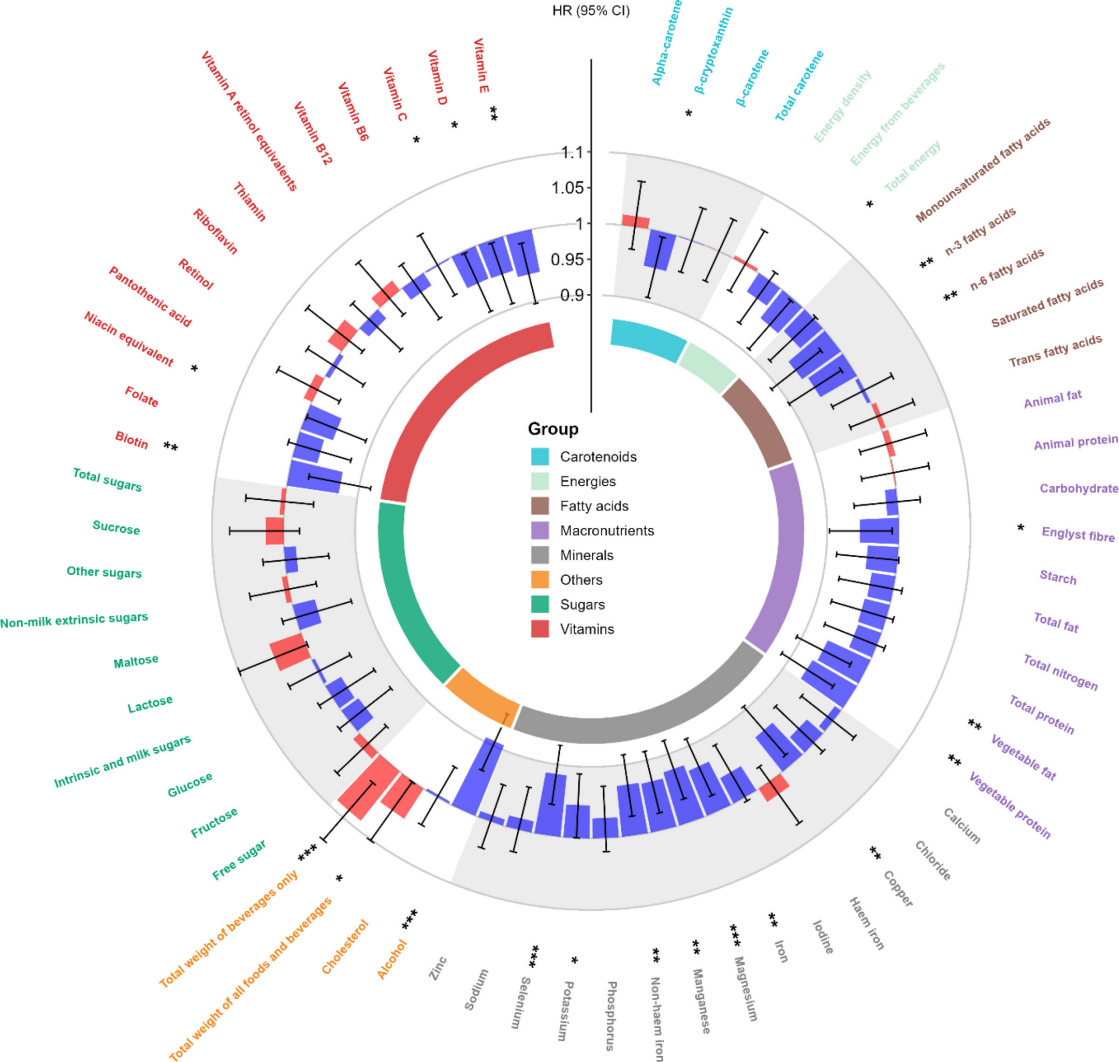
Associations between nutrients and RA risk. The circular plot displays hazard ratios (HRs) and 95% confidence intervals from Cox regression models for individual nutrients. Statistical models were adjusted for age, sex, ethnicity, education, TDI, smoking status, physical activity, sleep duration, hypertension, diabetes, and hyperlipidemia. Metabolites were grouped and color-coded by categories (carotenoids, fatty acids, macronutrients, minerals, etc.). Red denotes harmful effects and blue have protective effects. Statistical significance levels were indicated by asterisks (***P < 0.001, **P < 0.01, *P < 0.05).

After adjusting for confounding variables, 22 of the 63 nutrients were significantly associated with the incidence of RA (*P* < 0.05) (**Figure 1**). Among these nutrients, only total beverage intake and total food and beverage intake were positively associated with RA risk (HR = 1.08, 95% CI: 1.03–1.13; HR = 1.05, 95% CI: 1.00–1.10). In contrast, β-cryptoxanthin, total energy intake, n-3 fatty acids, n-6 fatty acids, Englyst fibre, vegetable fat, vegetable protein, copper, iron, magnesium, manganese, non-haem iron, potassium, selenium, alcohol, biotin, niacin equivalents, vitamin C, vitamin D, and vitamin E were inversely associated with RA risk. Detailed information can be found in **Supplementary Table 6**.

### 3.3 Association between nutrient risk score and RA risk

**Figure 2:**
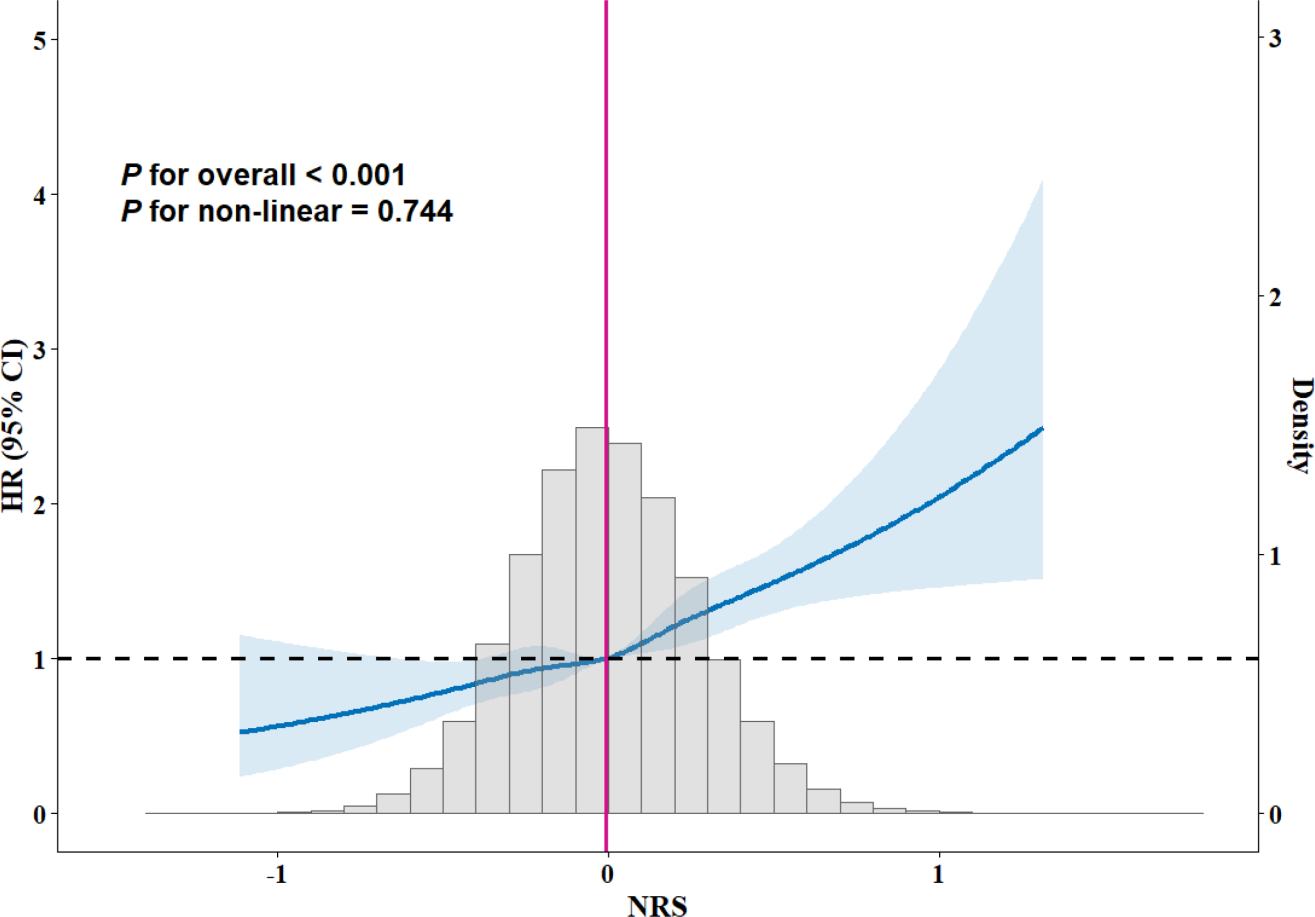
Restricted cubic spline of the associations between NRS and RA risk. Estimated effects were calculated using Cox models adjusted for age, sex, ethnicity, education, TDI, smoking status, physical activity, sleep duration, hypertension, diabetes, and hyperlipidemia.

**Table 2** presents the association between NRS and RA risk. After adjusting for multiple covariates (Model 3), each one-unit increment in NRS was associated with a 96% higher hazard of RA (HR = 1.96, 95% CI: 1.64-2.33, *P* < 0.001). Compared with participants in the lowest quartile (Q1), those in the highest quartile (Q4) of NRS had a significantly elevated risk of RA (HR = 1.53, 95% CI: 1.33-1.76; *P* < 0.001). In addition, no evidence of a nonlinear association was found between NRS and RA risk (*P* for nonlinearity > 0.05) (**Figure 2**).

**Table 2:** The associations between NRS and risk of RA.

NRS	Cases/N	Model 1		Model 3	
		HR (95% CI)	*P*-value	HR (95% CI)	*P*-value
Continuous	1,756/161,725	2.16 (1.81, 2.57)	<0.001	1.96 (1.64, 2.33)	<0.001
Quartile					
Q1	314/40,432	1 (Reference)		1 (Reference)	
Q2	368/40,431	1.05 (0.90, 1.22)	0.507	1.05 (0.91, 1.23)	0.502
Q3	460/40,431	1.25 (1.08, 1.44)	0.003	1.22 (1.06, 1.42)	0.007
Q4	614/40,431	1.62 (1.41, 1.86)	<0.001	1.53 (1.33, 1.76)	<0.001
*P* for trend			<0.001		<0.001

Model 1 adjusted for age, sex.

Model 2 adjusted model 1 + ethnicity, education, TDI, smoking status, physical activity, and sleep duration.

Model 3 adjusted model 2 + hypertension, diabetes, and hyperlipidemia.

CI, confidence interval; HR, hazard ratio; Q, quartile.

Models adjusted for the GPS showed results comparable to those from the Cox model. Higher NRS was significantly associated with an increased risk of RA (HR = 1.93, 95% CI: 1.61-2.32; *P* < 0.001). Consistent associations were observed in models weighted by IPW, irrespective of whether trimmed weights were applied (HR = 1.89, 95% CI: 1.54-2.32; *P* < 0.001 for untrimmed IPW; HR = 1.89, 95% CI: 1.58-2.27; *P* < 0.001 for trimmed IPW) (**Table 3**).

**Table 3:** Causal effect estimates of NRS on RA risk based on GPS and IPW methods.

Analysis	HR (95% CI)	*P*-value
Adjustment by GPS ^a^	1.93 (1.61, 2.32)	<0.001
Adjustment by IPW ^b^	1.89 (1.54, 2.32)	<0.001
Adjustment by IPW ^c^	1.89 (1.58, 2.27)	<0.001

All models were adjusted for age, sex, ethnicity, education, TDI, smoking status, physical activity, sleep duration, hypertension, diabetes, and hyperlipidemia.

a: Adjusting for GPS as variable;.

b: Based on the original GPS distribution;.

c: Trimmed IPWs based on 1st and 99th percentile of the GPS distribution.

**Figure 3:**
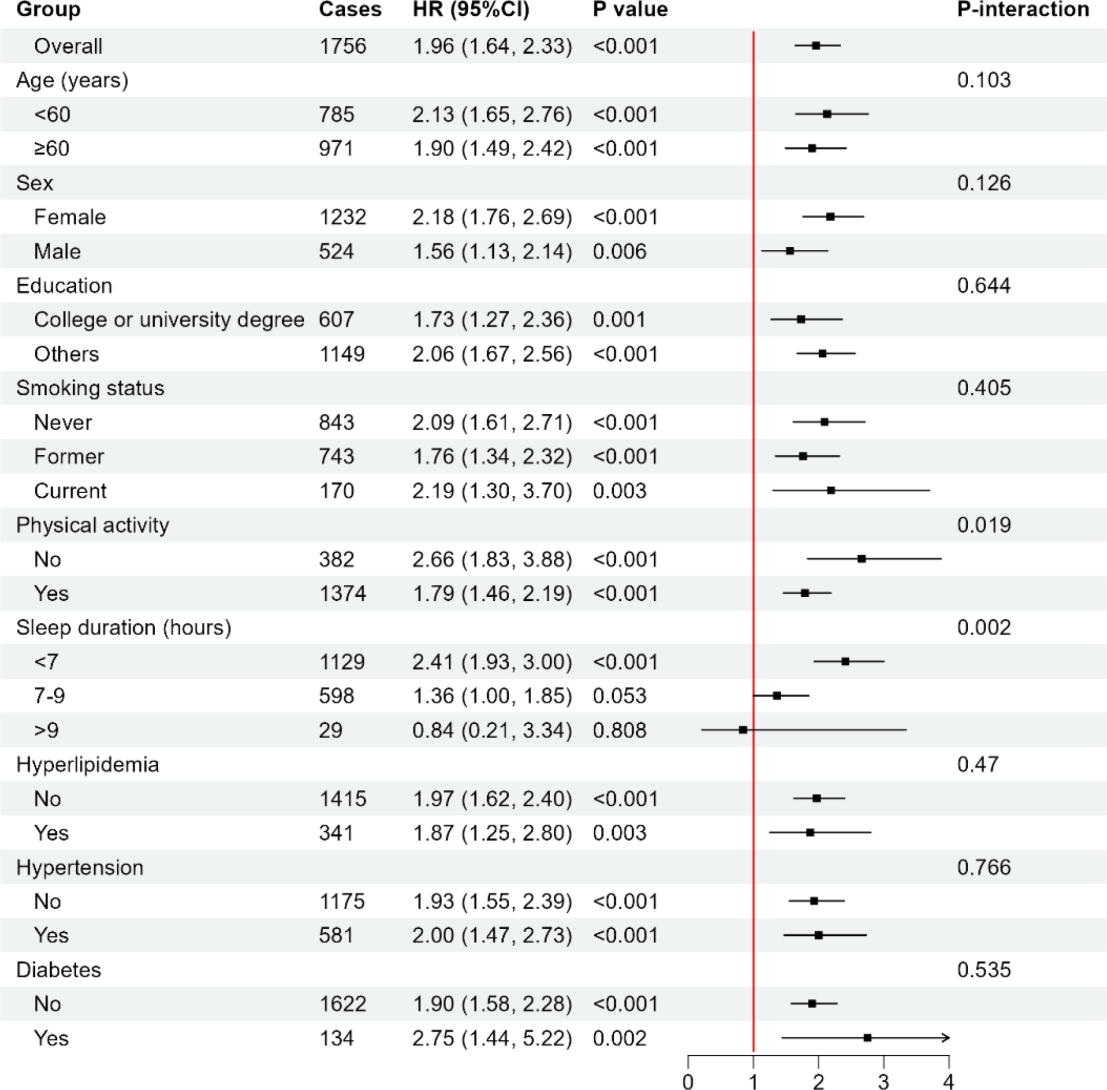
Subgroup analyses of the association between NRS and RA risk. Models were adjusted for age, sex, ethnicity, education, TDI, smoking status, physical activity, sleep duration, hypertension, diabetes, and hyperlipidemia.

The positive association between NRS and RA risk was generally consistent across subgroups, but was not statistically significant among individuals with sleep durations of 7–9 or ≥9 hours (**Figure 3**). Notably, stronger associations were observed among individuals with insufficient physical activity and short sleep duration (<7 hours), with significant multiplicative interactions detected for physical activity (*P* = 0.019) and sleep duration (*P* = 0.002).

To assess the robustness of our findings, we conducted several additional sensitivity analyses (**Supplementary Tables 7**-**9**). The associations remained statistically significant after excluding participants with missing baseline data. Comparable results were obtained after removing individuals who developed RA within the first two years of follow-up. Furthermore, analyses based on competing risk models treating death as a competing event yielded materially unchanged estimates.

### 3.4 Combined effects of NRS and genetic susceptibility

We initially examined the association between genetic susceptibility and RA incidence (**Supplementary Table 10**). Each unit increase in the PRS was associated with a 34% higher risk of incident RA (HR = 1.34, 95% CI: 1.28-1.41). Individuals at intermediate and high genetic risk exhibited a 20% (95% CI: 6%-36%) and 87% (95% CI: 67%-110%) greater risk of RA, respectively, compared with those at low genetic risk. Furthermore, joint analysis of NRS and PRS demonstrated that participants in the highest categories of both genetic risk and NRS had more than a threefold increased risk of RA relative to those in the lowest categories (HR = 3.28, 95% CI: 2.49–4.32) (**Table 4**). Although no significant additive interaction between NRS and genetic susceptibility was observed (*P*_Additive interaction_ > 0.05) (**Supplementary Table 11**), a statistically significant multiplicative interaction was identified (*P*_Multiplicative interaction_ = 0.005) (**Supplementary Table 12**).

**Table 4:** Association of NRS and genetic risk with incident RA.

PRS & NRS	Cases/N	HR (95% CI)	*P*-value	*P* for trend
Low genetic risk				<0.001
Q1	64/13,560	1 (Reference)		
Q2	87/13,530	1.24 (0.90, 1.71)	0.197	
Q3	108/13,505	1.43 (1.05, 1.95)	0.023	
Q4	171/13,314	2.16 (1.61, 2.88)	<0.001	
Medium genetic risk				<0.001
Q1	80/13,355	1.28 (0.92, 1.78)	0.135	
Q2	116/13,527	1.65 (1.21, 2.23)	0.001	
Q3	143/13,416	1.90 (1.42, 2.56)	<0.001	
Q4	178/13,610	2.18 (1.64, 2.91)	<0.001	
High genetic risk				0.007
Q1	170/13,517	2.68 (2.01, 3.58)	<0.001	
Q2	165/13,374	2.35 (1.76, 3.14)	<0.001	
Q3	209/13,510	2.75 (2.07, 3.14)	<0.001	
Q4	265/13,507	3.28 (2.49, 4.32)	<0.001	

Models were adjusted for age, sex, ethnicity, education, TDI, smoking status, physical activity, sleep duration, hypertension, diabetes, and hyperlipidemia.

### 3.5 Mediation analysis

In the mediation analysis, 10 obesity-related indicators were found to play mediating roles in the association between NRS and RA risk (**Table 5**). Among them, WHtR showed the strongest mediating effect, accounting for 13.57% of the association. In addition, BMI, WC, BRI, LAP, VAI, TyG-BMI, TyG-WC, TyG-WHtR, and BF% mediated 13.22%, 10.69%, 12.83%, 5.62%, 3.81%, 11.77%, 9.14%, 11.33%, and 12.84% of the association between NRS and RA risk, respectively.

**Table 5:** Mediation analysis of obesity on associations of NRS with incident RA.

Obesity indices	Total effect	Natural direct effect	Natural indirect effect	Proportion mediated, % (95% CI)
	HR (95% CI)	HR (95% CI)	HR (95% CI)	
Body shape				
BMI	1.93 (1.63, 2.29) ^*^	1.80 (1.52, 2.15) ^*^	1.07 (1.05, 1.08) ^*^	13.22 (9.63, 17.79) ^*^
WC	1.93 (1.63, 2.30) ^*^	1.84 (1.54, 2.18) ^*^	1.05 (1.04, 1.07) ^*^	10.69 (7.92, 14.62) ^*^
WHtR	1.93 (1.63, 2.29) ^*^	1.81 (1.52, 2.15) ^*^	1.07 (1.05, 1.09) ^*^	13.57 (10.29, 18.67) ^*^
BRI	1.93 (1.61, 2.29) ^*^	1.81 (1.51, 2.16) ^*^	1.07 (1.05, 1.08) ^*^	12.83 (9.38, 17.45) ^*^
ABSI	1.95 (1.65, 2.32) ^*^	1.94 (1.64, 2.31) ^*^	1.00 (1.00, 1.00)	0.25 (-0.05, 0.67)
Visceral fat accumulation				
LAP	1.95 (1.63, 2.31) ^*^	1.89 (1.58, 2.25) ^*^	1.03 (1.02, 1.04) ^*^	5.62 (3.56, 8.36) ^*^
VAI	1.95 (1.64, 2.31) ^*^	1.91 (1.61, 2.27) ^*^	1.02 (1.01, 1.03) ^*^	3.81 (1.77, 6.11) ^*^
Metabolic function				
TyG	1.95 (1.63, 2.31) ^*^	1.95 (1.63, 2.31) ^*^	1.00 (0.99, 1.00)	-0.08 (-1.12, 0.84)
TyG-BMI	1.93 (1.60, 2.30) ^*^	1.82 (1.51, 2.17) ^*^	1.06 (1.04, 1.08) ^*^	11.77 (8.57, 15.61) ^*^
TyG-WC	1.94 (1.64, 2.31) ^*^	1.86 (1.56, 2.20) ^*^	1.05 (1.03, 1.06) ^*^	9.14 (6.42, 13.03) ^*^
TyG-WHtR	1.94 (1.64, 2.30) ^*^	1.83 (1.54, 2.19) ^*^	1.06 (1.04, 1.07) ^*^	11.33 (8.13, 15.71) ^*^
BF%	1.94 (1.63, 2.30) ^*^	1.82 (1.52, 2.17) ^*^	1.07 (1.05, 1.09) ^*^	12.84 (8.53, 18.40) ^*^

Models adjusted for age, sex, ethnicity, education, TDI, smoking status, physical activity, sleep duration, hypertension, diabetes, and hyperlipidemia. Statistical significance levels were indicated by asterisks (*P < 0.05).

## 4 Discussion

To our knowledge, this is the first prospective cohort study investigating the association between multiple nutrient intakes and RA risk. We constructed a NRS based on 14 nutrients to reflect the overall nutrient exposure profile and systematically evaluated its relationship with RA risk. We further examined the modifying effect of genetic susceptibility and the mediating role of obesity. The results showed a significant positive association between NRS and RA risk. Although this association was attenuated among individuals with high genetic risk, those with both high NRS and high genetic susceptibility still had a markedly increased RA risk. In addition, the adverse effect of NRS on RA risk was partly mediated through obesity.

The findings add to the current understanding of how nutrient intake relates to RA. The NRS captures the combined influence of multiple nutrients included in the score, several of which are directly involved in key biological pathways related to RA pathogenesis. Specifically, nutrients with negative coefficients in the NRS, such as β-carotene, β-cryptoxanthin, selenium, vegetable protein, monounsaturated fatty acids, niacin equivalent, potassium, and biotin, are known to exert anti-inflammatory, antioxidant, and immunomodulatory effects. Among these, carotenoids (β-carotene and β-cryptoxanthin) and selenium play important roles in reducing oxidative stress and regulating immune responses, while monounsaturated fatty acids and plant-derived nutrients may attenuate systemic inflammation. In contrast, nutrients with positive coefficients, including cholesterol, sodium, haem iron, and pantothenic acid, may contribute to pro-inflammatory processes, oxidative stress, or metabolic dysregulation, thereby potentially increasing RA risk (34–36). These pathways may act synergistically, and their joint effects could better reflect the complex role of diet in RA development than analyses focusing on individual nutrients. Previous observational and experimental studies have explored the associations between various nutrients and RA risk. Specifically, lutein has been shown to slow RA progression by suppressing inflammatory responses and preserving joint structural integrity (37). Several population-based cohort studies have reported potential protective effects of n-3 polyunsaturated fatty acid intake against RA development (10, 38). Intake of one-carbon metabolism nutrients may reduce RA risk through several biological mechanisms, such as maintaining DNA methylation homeostasis, inhibiting the abnormal invasiveness of synovial fibroblasts, modulating immune responses, and influencing ferroptosis pathways (7, 39). In addition, dietary iron, magnesium, phosphorus, and copper may exert protective effects via immune regulation, anti-inflammatory actions, oxidative stress control, and maintenance of bone metabolic homeostasis (40–42). Similar directional associations between these nutrients and RA risk were observed in our study, but they were not statistically significant, likely due to differences in model selection, confounding factors adjustment, and nutrient handling.

Notably, our study identified a significant positive association between total beverage consumption and incident RA risk, suggesting that overall beverage intake patterns may play a role in RA development. Higher beverage consumption is often accompanied by increased intake of sugar-sweetened beverages, fruit juices, and coffee, which may contribute to adverse metabolic and inflammatory profiles. Excessive sugar intake can induce insulin resistance, promote oxidative stress, and activate pro-inflammatory signaling pathways, thereby exacerbating systemic inflammation (34). In addition, beverages have a relatively low satiety effect and can easily lead to weight gain. Obesity and related metabolic abnormalities are important risk factors for RA and may aggravate systemic inflammation by increasing the production of inflammatory adipokines from adipose tissue (43). Moreover, sugars and caffeine present in beverages could disturb intestinal microbial equilibrium, compromise immune regulation, and facilitate autoimmune activation (34). Overall, higher total beverage consumption may increase the likelihood of developing RA through the interplay of multiple inflammatory, metabolic, and immune mechanisms. Previous epidemiological studies have reported an inverse association between moderate alcohol intake and the risk of rheumatoid arthritis (RA) (44–46), consistent with our finding of a negative association between alcohol intake and RA risk in the NRS. However, this result should be interpreted cautiously, as repeated measurements may attenuate extreme intake and residual confounding (e.g., alcohol type and drinking patterns) cannot be excluded. Biologically, moderate alcohol consumption may exert anti-inflammatory and immunomodulatory effects, whereas excessive intake promotes systemic inflammation and immune dysfunction, indicating a dose-dependent and context-specific relationship. Overall, these results highlight the complex and heterogeneous effects of different beverage components on RA risk, warranting further investigation into specific beverage types and underlying mechanisms.

Compared with previous studies, this prospective cohort study not only systematically evaluated the associations between multiple nutrients and RA risk but also constructed a NRS to more comprehensively capture the relationship between overall nutritional status and RA risk. In addition, GPS and IPW methods were applied to balance baseline covariates across exposure levels, thereby controlling for confounding and improving the robustness of the results.

The present study demonstrates a clear role of genetic factors in RA susceptibility, corroborating prior research (47). In addition, previous studies suggest that individuals with high genetic risk who consume higher amounts of one-carbon metabolism nutrients and polyunsaturated fatty acids may have a lower risk of RA (7, 10). Although interactions between genetic risk and individual nutrients have been explored to some extent, evidence regarding the interplay between genetic susceptibility and overall nutrient intake patterns in the development of RA remains limited. Our study showed that the association between NRS and RA risk varied across genetic risk levels, with a significant multiplicative interaction, indicating that the relative effect of nutrient exposure differs by genetic susceptibility. This pattern may reflect interactions between genetic predisposition and environmentally driven inflammatory and metabolic pathways. However, no significant additive interaction was observed in our study, suggesting limited evidence of a joint effect on an absolute risk scale. Overall, individuals with both higher NRS and higher genetic susceptibility tended to have higher risk estimates, supporting the potential value of integrating genetic and nutritional information for risk stratification.

In addition, we observed that the association between NRS and RA risk was partly mediated by multiple obesity-related indicators. Compared with other central obesity measures, WHtR can better reflect abdominal fat accumulation and provide a more accurate assessment of visceral fat distribution and body shape, thereby offering superior prediction of obesity-related health risks (48, 49). Moreover, WHtR is less affected by sex and racial differences (50), and has been recommended by UK NICE guidelines as an alternative indicator for obesity assessment (51). Other commonly used indices of body shape and visceral fat accumulation, including BMI, WC, BRI, LAP, VAI, and BF%, also showed varying degrees of mediating effects, suggesting that both general and abdominal obesity contribute to the association between NRS and RA risk. Notably, although several TyG-derived indices (TyG-BMI, TyG-WC, and TyG-WHtR) showed significant mediated effects, the TyG index itself did not. This pattern suggests that the observed mediation in our study may be more strongly driven by body composition and fat distribution rather than insulin resistance alone. From a biological perspective, obesity is closely linked to chronic low-grade inflammation, dysregulated adipokine secretion, and immune activation, which can act as intermediate pathways linking nutrient intake to RA development. Overall, these observations indicate that the influence of NRS on RA risk may be partially mediated by obesity, emphasizing the importance of maintaining healthy body weight to reduce the detrimental effects associated with high NRS.

Our study has a number of advantages. First, it is an important prospective study examining the longitudinal relationship between NRS and RA risk in a large population-based sample with long follow-up period, which substantially increases the statistical power and reliability of the results. Second, the robustness of the findings was supported by subgroup analyses, a series of sensitivity analyses, and adjustment for potential confounders using GPS and IPW. Third, we explored the interaction effect of PRS on the association between NRS and RA risk, and the further adjustment of genetic risk in the main analysis strengthens the validity of our findings. Lastly, we explored the role of obesity as a mediator between NRS and RA risk, providing additional insights for RA prevention.

There are still several limitations that should be noted. First, dietary intake was self-reported, which can lead to recall bias, day-to-day variability, and measurement error. Although repeated assessments were averaged to better reflect habitual intake, some degree of misclassification cannot be excluded, and the use of short-term dietary measures may introduce within-person variability, potentially leading to regression dilution bias and attenuating the observed associations. Second, we only considered nutrients at baseline and did not account for their dynamic changes during the follow-up period, therefore the impact of the longitudinal changes in nutrient intake on RA risk could not be estimated. Third, despite the application of GPS and IPW to control for measured confounding, the observational design inherently limits causal inference, and residual confounding from unmeasured or imperfectly measured factors cannot be fully excluded. Fourth, the mediation and interaction analyses rely on assumptions that cannot be fully verified in observational studies, and the resulting estimates may be influenced. Finally, the UK Biobank is not a fully representative sample of the general population, as participants are predominantly of White ethnicity and tend to be healthier, better educated, and more health-conscious than the general population, which may introduce selection bias and healthy volunteer bias. These factors could lead to underestimation of disease incidence and potentially attenuate observed associations. Moreover, differences in genetic background, dietary patterns, and environmental exposures across populations may limit the generalizability of our findings to other ethnic groups and regions. Therefore, caution is warranted when extrapolating these results to global RA epidemiology, and further studies in more diverse populations are needed to validate our findings.

## 5 Conclusions

In conclusion, our study reveals a significant association between NRS and RA risk. Individuals exhibiting both high NRS and elevated genetic susceptibility faced the highest likelihood of developing RA. The significant mediating roles of multiple obesity-related indicators in the association between the NRS and RA provide mechanistic insights into RA from an epidemiological perspective. Mitigating obesity may help attenuate the adverse effects of a high NRS on RA risk. These findings highlight the potential clinical value of the NRS as a novel risk stratification tool for RA and provide a scientific basis for developing targeted dietary strategies for high-risk populations.

## Data Availability

Publicly available datasets were analyzed in this study. This data can be found here: www.ukbiobank.ac.uk.
