# Supplementary material for: Association of a data-driven nutrient risk score with incident rheumatoid arthritis in UK Biobank adults: a prospective cohort study
Source: Front Immunol. 2026 May 26;17:1831599. doi: 10.3389/fimmu.2026.1831599 (PMC13246429; doi:10.3389/fimmu.2026.1831599)
Supplement: Supplementary file 1 [file DataSheet1.docx]

Supplementary Material

# Graphical abstract


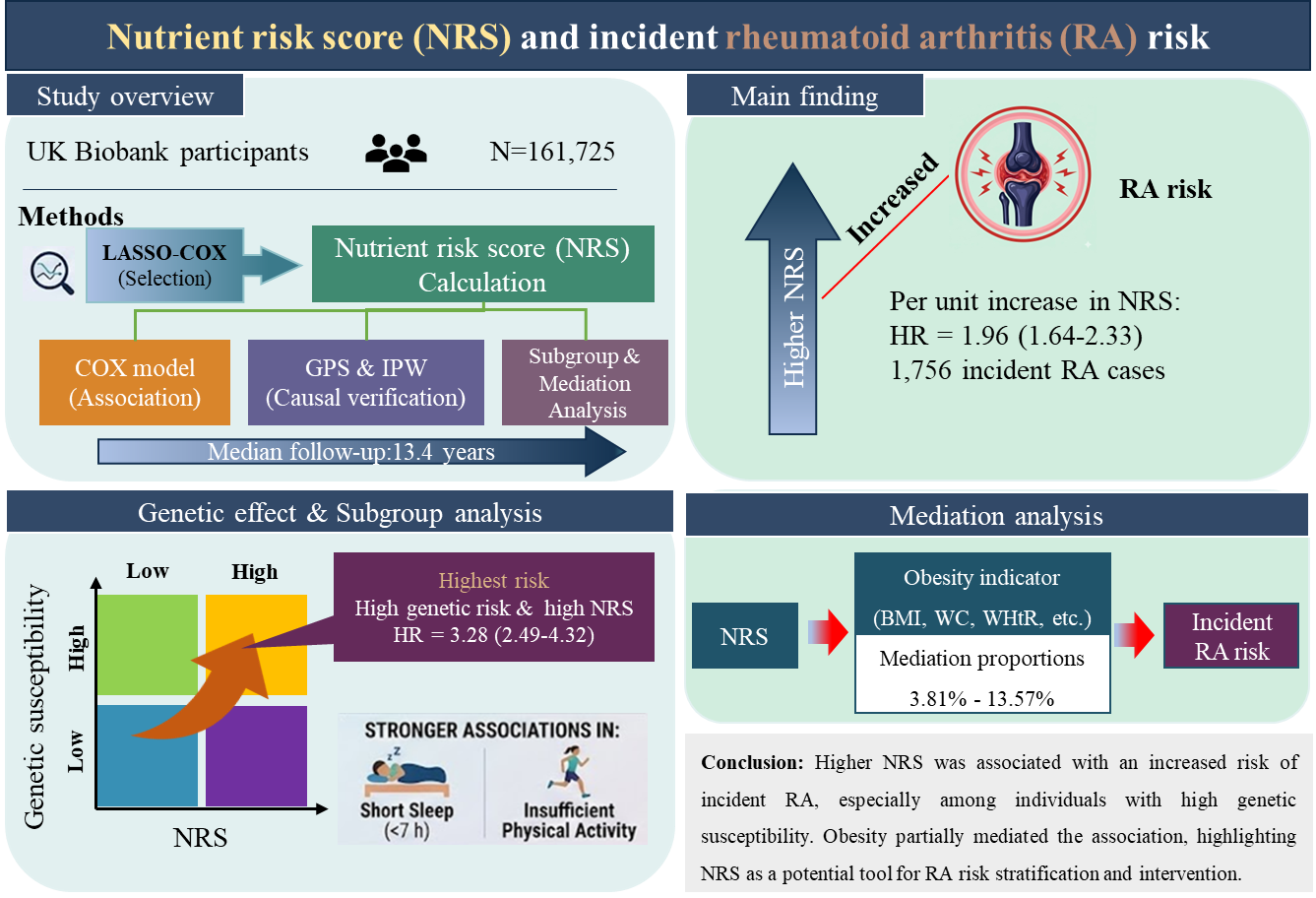


# Supplementary Figures and Tables

## Supplementary Figures


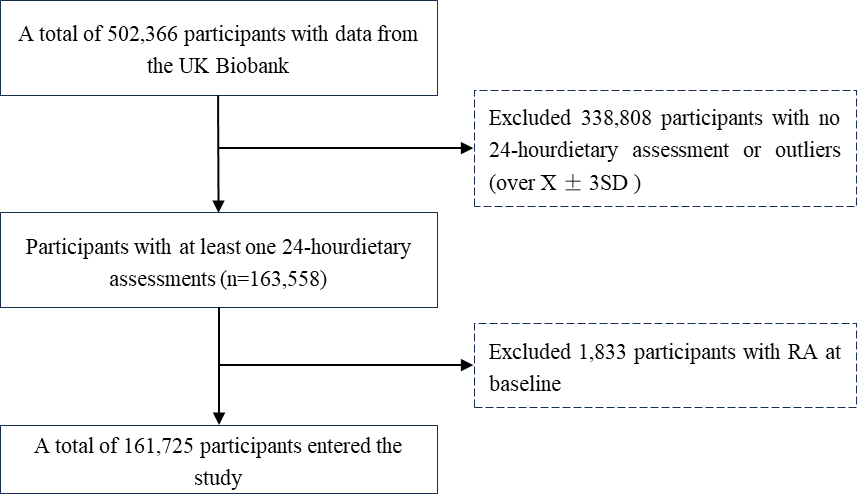


**Supplementary Figure 1.** Flow chart of inclusion-exclusion criteria for UKB study subjects.

**
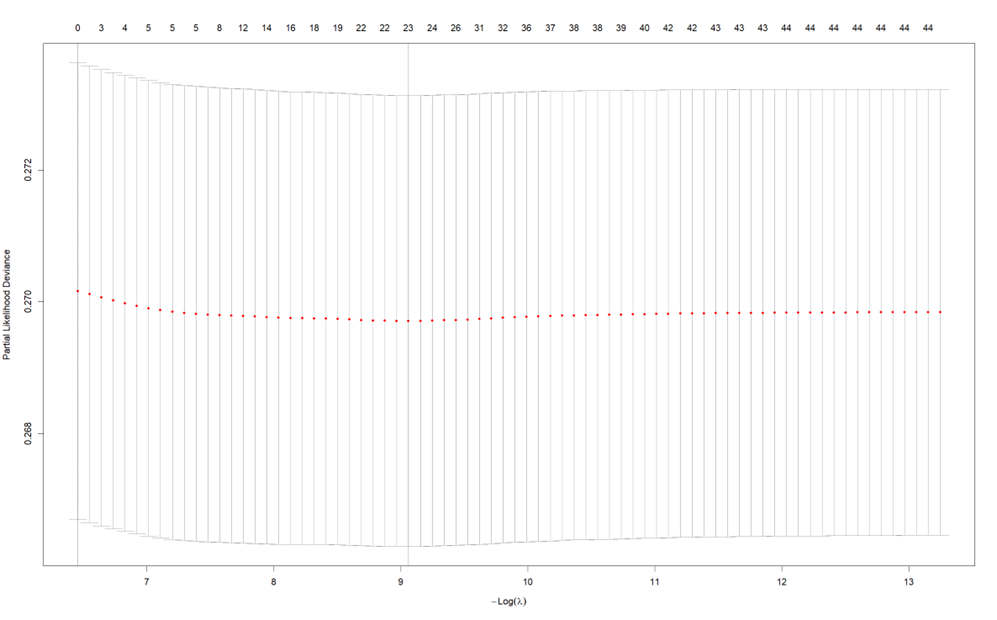
**

**Supplementary Figure 2.** Cross-validation curve for LASSO-Cox regression model selection.

**
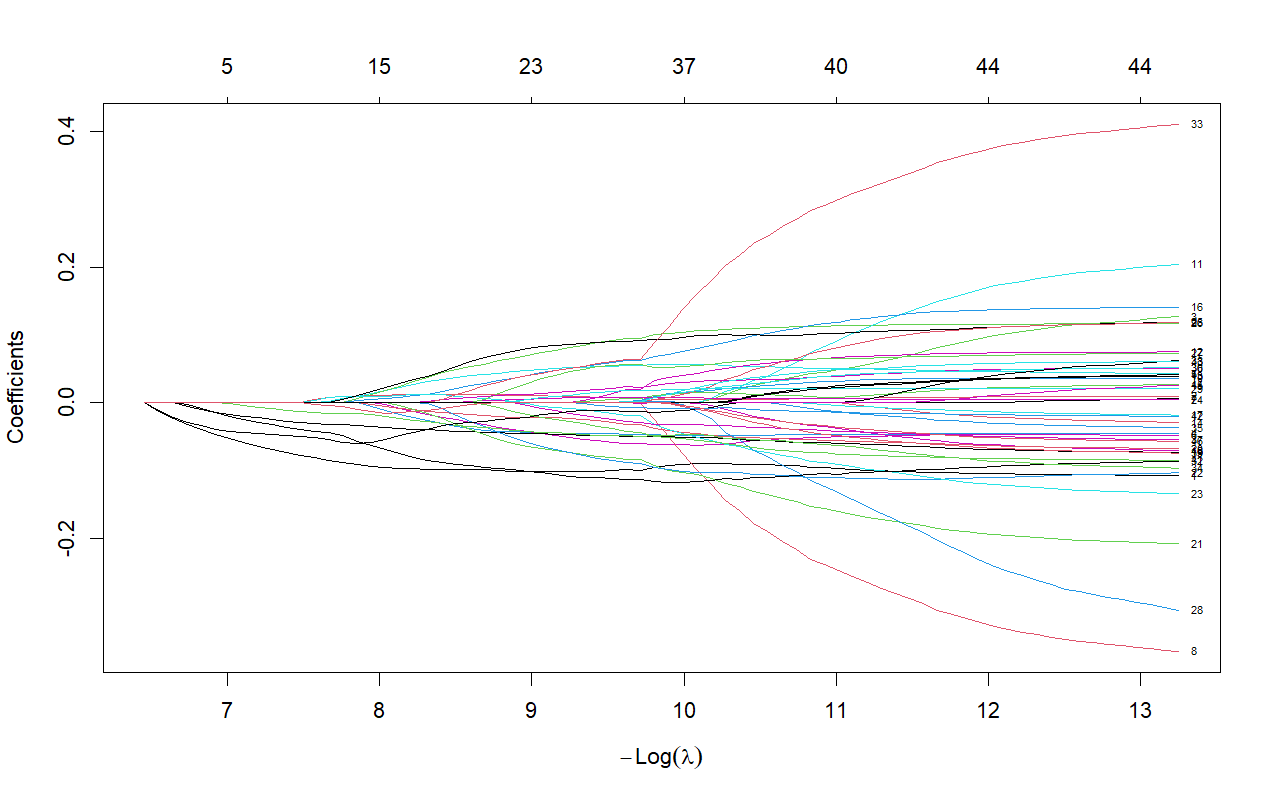
**

**Supplementary Figure 3.** LASSO coefficient path for nutrients.


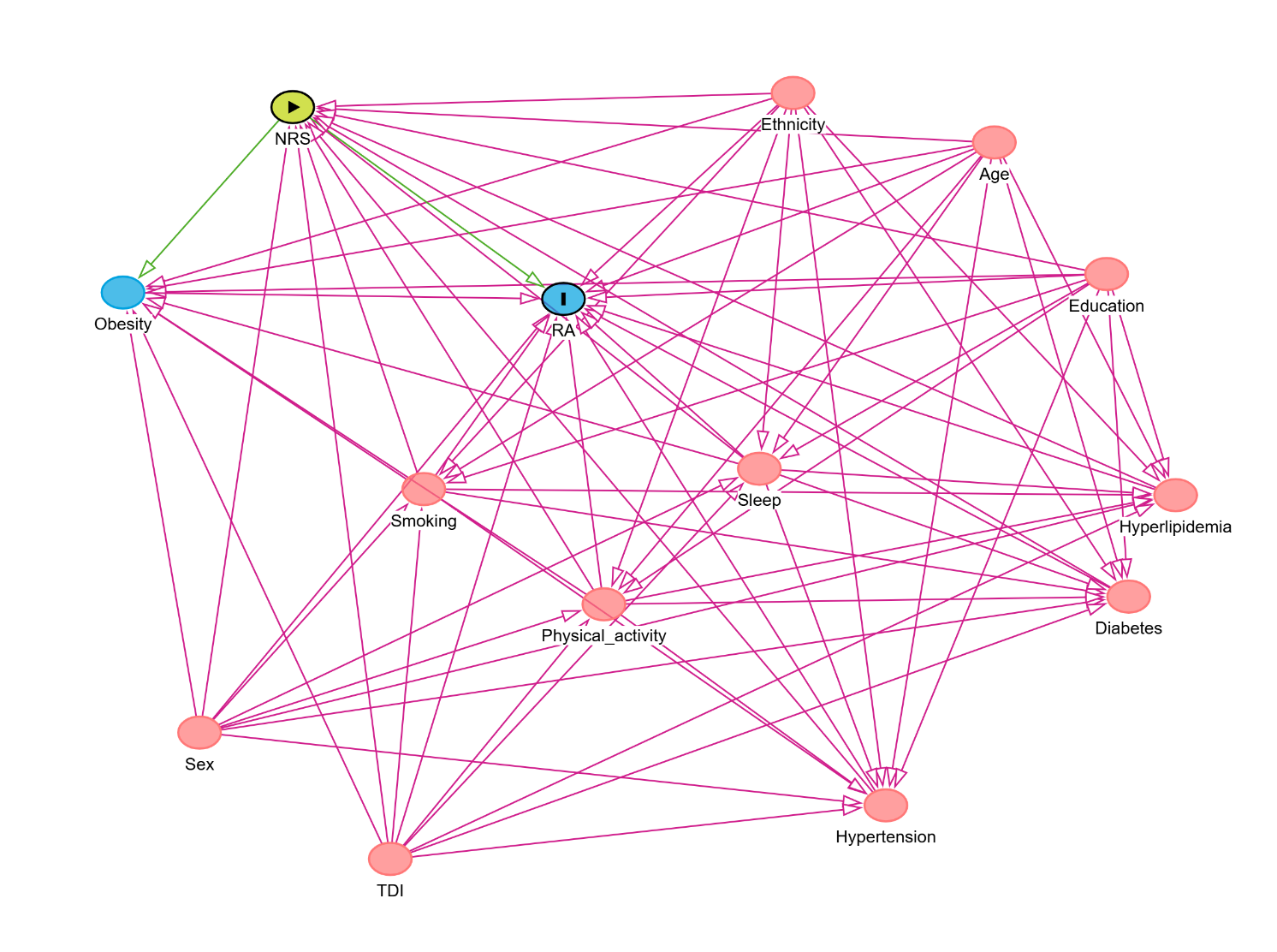


**Supplementary Figure 4.** The directed acyclic graph based on causal hypothesis.

## Supplementary Tables

**Supplementary Table 1 List of nutrient names and groups in the UK Biobank**

| Nutrients | Groups | Unit | Field ID |
| --- | --- | --- | --- |
| Alpha-carotene | Carotenoids | ug | 26038 |
| β-cryptoxanthin | Carotenoids | ug | 26040 |
| β-carotene | Carotenoids | ug | 26039 |
| Total carotene | Carotenoids | ug | 26027 |
| Monounsaturated fatty acids | Fatty acids | g | 26032 |
| Saturated fatty acids | Fatty acids | g | 26014 |
| Trans fatty acids | Fatty acids | g | 26155 |
| n-3 fatty acids | Fatty acids | g | 26015 |
| n-6 fatty acids | Fatty acids | g | 26016 |
| Animal fat | Macronutrients | g | 26010 |
| Animal protein | Macronutrients | g | 26007 |
| Carbohydrate | Macronutrients | g | 26013 |
| Englyst fibre | Macronutrients | g | 26017 |
| Starch | Macronutrients | g | 26031 |
| Total fat | Macronutrients | g | 26008 |
| Total protein | Macronutrients | g | 26005 |
| Total nitrogen | Macronutrients | g | 26060 |
| Vegetable fat | Macronutrients | g | 26009 |
| Vegetable protein | Macronutrients | g | 26006 |
| Calcium | Minerals | mg | 26018 |
| Chloride | Minerals | mg | 26042 |
| Copper | Minerals | mg | 26043 |
| Haem iron | Minerals | mg | 26046 |
| Iodine | Minerals | ug | 26047 |
| Iron | Minerals | mg | 26019 |
| Magnesium | Minerals | mg | 26025 |
| Manganese | Minerals | mg | 26051 |
| Non-haem iron | Minerals | mg | 26053 |
| Phosphorus | Minerals | mg | 26036 |
| Potassium | Minerals | mg | 26024 |
| Selenium | Minerals | ug | 26058 |
| Sodium | Minerals | mg | 26052 |
| Zinc | Minerals | mg | 26033 |
| Total energy | Energies | kj | 26002 |
| Energy density | Energies | kj/g | 26004 |
| Energy from beverages | Energies | kj | 26003 |
| Alcohol | Others | g | 26030 |
| Cholesterol | Others | mg | 26037 |
| Total weight of all foods and beverages | Others | g | 26000 |
| Total weight of beverages only | Others | g | 26001 |
| Free sugar | Sugars | g | 26012 |
| Fructose | Sugars | g | 26044 |
| Glucose | Sugars | g | 26045 |
| Intrinsic and milk sugars | Sugars | g | 26050 |
| Lactose | Sugars | g | 26048 |
| Maltose | Sugars | g | 26049 |
| Non-milk extrinsic sugars | Sugars | g | 26055 |
| Other Sugars | Sugars | g | 26056 |
| Sucrose | Sugars | g | 26059 |
| Total sugars | Sugars | g | 26011 |
| Biotin | Vitamins | ug | 26041 |
| Folate | Vitamins | ug | 26022 |
| Niacin equivalent | Vitamins | mg | 26054 |
| Pantothenic acid | Vitamins | mg | 26057 |
| Retinol | Vitamins | ug | 26026 |
| Riboflavin | Vitamins | mg | 26035 |
| Thiamin | Vitamins | mg | 26034 |
| Vitamin A retinol equivalents | Vitamins | ug | 26061 |
| Vitamin B12 | Vitamins | ug | 26021 |
| Vitamin B6 | Vitamins | mg | 26020 |
| Vitamin C | Vitamins | mg | 26023 |
| Vitamin D | Vitamins | ug | 26029 |
| Vitamin E | Vitamins | mg | 26028 |

**Supplementary Table 2 Nutrients associated with RA were identified using a LASSO-Cox model.**

| Nutrients | Coef | Frequency (%) |
| --- | --- | --- |
| Alcohol | -0.101396774 | 0.99 |
| β-cryptoxanthin | -0.043951531 | 0.89 |
| β-carotene | 0.048605636 | 0.84 |
| Biotin | -0.045524188 | 0.91 |
| Cholesterol | 0.073463918 | 0.99 |
| Haem iron | 0.043807565 | 0.96 |
| Maltose | -0.046050928 | 0.97 |
| Manganese | -0.02239584 | 0.83 |
| Monounsaturated fatty acids | -0.068107682 | 0.92 |
| Niacin equivalent | -0.064618904 | 0.89 |
| Other sugars | 0.006114733 | 0.83 |
| Pantothenic acid | 0.08243702 | 0.93 |
| Potassium | 0.034989777 | 0.82 |
| Retinol | 0.006987479 | 0.88 |
| Selenium | -0.103462913 | 0.99 |
| Sodium | 0.043719069 | 0.99 |
| Starch | -0.043460012 | 0.85 |
| Trans fatty acids | -0.0082105 | 0.86 |
| Vegetable protein | -0.01800511 | 0.81 |
| Vitamin C | -0.021077633 | 0.87 |

**Supplementary Table 3 Associations between 12 nutrients used in NRS and RA risk**

| Nutrients | Coef | *HR* (95% *CI*) | *P*-value |
| --- | --- | --- | --- |
| Alcohol | -0.11 | 0.90 (0.85, 0.94) | <0.001 |
| Vegetable protein | -0.07 | 0.93 (0.86, 1.01) | 0.069 |
| β-carotene | 0.08 | 1.08 (1.02, 1.15) | 0.014 |
| Selenium | -0.11 | 0.89 (0.83, 0.96) | 0.002 |
| Pantothenic acid | 0.10 | 1.11 (1.01, 1.21) | 0.023 |
| β-cryptoxanthin | -0.08 | 0.92 (0.87, 0.98) | 0.007 |
| Maltose | -0.05 | 0.95 (0.87, 1.01) | 0.090 |
| Cholesterol | 0.11 | 1.12 (1.04, 1.20) | 0.004 |
| Monounsaturated fatty acids | -0.12 | 0.89 (0.83, 0.95) | 0.001 |
| Sodium | 0.09 | 1.09 (1.02, 1.17) | 0.013 |
| Haem iron | 0.07 | 1.07 (1.01, 1.14) | 0.031 |
| Niacin equivalent | -0.13 | 0.88 (0.80, 0.97) | 0.013 |
| Potassium | 0.08 | 1.08 (0.99, 1.19) | 0.098 |
| Biotin | -0.06 | 0.95 (0.88, 1.02) | 0.148 |

**Supplementary Table 4.** **Calculation methods of combination in each obesity indices**

| Indicators | Definition or calculation formula |
| --- | --- |
| BMI | $weight(kg)/ {[height(m)]}^{2}$ |
| WHtR | $WC(cm)/ height(cm)$ |
| BRI | $364.2-365.5\times\sqrt{(1-[WC(cm)\div2\pi)]^{2})\div\left[ 0.5\times Height(cm) \right]^{2}}$ |
| ABSI | $\mathrm{ABSI}=1000\times\mathrm{WC}\left( m \right)\times\mathrm{Weight}\left( \mathrm{kg} \right)^{-2/3}\times\mathrm{Height}\left( m \right)^{5/6}$ |
| VAI | $\mathrm{VAI}=\left( \frac{\mathrm{WC}\left( \mathrm{cm} \right)}{39.68+1.88\times\mathrm{BMI}\left( kg/m^{2} \right)} \right)\times\left( \frac{\mathrm{TG}\left( mmol/L \right)}{1.03} \right)\times\left( \frac{1.31}{HDL-C\left( mmol/L \right)} \right)$ for male  $\mathrm{VAI}\begin{aligned} =\left( \frac{\mathrm{WC}\left( \mathrm{cm} \right)}{36.58+1.89\times\mathrm{BMI}\left( kg/m^{2} \right)} \right) \end{aligned}\times\left( \frac{\mathrm{TG}\left( mmol/L \right)}{0.81} \right)\times\left( \frac{1.52}{HDL-C\left( mmol/L \right)} \right)$ for female |
| LAP | $[WC (cm)-65]\times TG(mmol/L)$ for male  $[WC (cm)-58]\times TG(mmol/L)$ for female |
| TyG | $ln(\frac{TG(mg/dL)*FPG(mg/dL)}{2})$ |
| TyG-BMI | $TyG*BMI$ |
| TyG-WC | $TyG*WC$ |
| TyG- WHtR | TyG*WHtR |
| BF% | was measured via bioelectrical impedance analysis, performed with the Tanita BC418MA Body Composition Analyzer |

Abbreviations: BMI, body mass index; WC, waist circumference; WHtR, waist-to-height ratio; BRI, body roundness index; ABSI, a body shape index; TG, triglyceride; HDL-C, high-density lipoprotein cholesterol; VAI, visceral adiposity index; LAP, lipid accumulation product; FBG, fasting blood glucose; TyG, triglyceride-glucose index; BF%, body fat percentage.

**Supplementary Table 5. Missing rates for variables used in the analysis**

| Variables | Missing Rate (%) |
| --- | --- |
| Ethnicity | 0.35 |
| Education | 0.47 |
| Smoking status | 0.25 |
| Physical activity | 17.63 |
| Sleep duration | 0.31 |
| TDI | 0.94 |
| BMI | 0.28 |
| WC | 0.16 |
| BF% | 1.56 |
| ABSI | 0.30 |
| WHtR | 0.23 |
| BRI | 0.23 |
| LAP | 5.79 |
| VAI | 14.12 |
| TyG | 13.95 |
| TyG-BMI | 14.16 |
| TyG-WC | 14.06 |
| TyG-WHtR | 14.12 |
| PRS | 2.28 |

Abbreviations: TDI, Townsend deprivation index; BMI, body mass index; WC, waist circumference; BF%, body fat percentage; ABSI, a body shape index; WHtR, waist-to-height ratio; BRI, body roundness index; LAP, lipid accumulation product; VAI, visceral adiposity index; TyG, triglyceride-glucose index; PRS, polygenic risk score.

**Supplementary Table 6. Associations between nutrients and RA risk**

| Nutrients | Group | *HR* (95% *CI*) | *P*-value |
| --- | --- | --- | --- |
| Alpha-carotene | Carotenoids | 1.01 (0.97, 1.06) | 0.543 |
| β-cryptoxanthin | Carotenoids | 0.95 (0.91, 0.99) | 0.023 |
| β-carotene | Carotenoids | 1.00 (0.95, 1.05) | 0.971 |
| Total carotene | Carotenoids | 1.00 (0.95, 1.05) | 0.990 |
| Energy density | Energies | 1.00 (0.96, 1.05) | 0.849 |
| Energy from beverages | Energies | 0.97 (0.93, 1.01) | 0.171 |
| Total energy | Energies | 0.95 (0.91, 1.00) | 0.034 |
| Monounsaturated fatty acids | Fatty acids | 0.96 (0.91, 1.00) | 0.069 |
| n-3 fatty acids | Fatty acids | 0.93 (0.89, 0.98) | 0.004 |
| n-6 fatty acids | Fatty acids | 0.94 (0.90, 0.99) | 0.010 |
| Saturated fatty acids | Fatty acids | 1.00 (0.95, 1.04) | 0.835 |
| Trans fatty acids | Fatty acids | 1.01 (0.96, 1.06) | 0.788 |
| Animal fat | Macronutrients | 1.01 (0.96, 1.06) | 0.726 |
| Animal protein | Macronutrients | 1.00 (0.95, 1.05) | 0.949 |
| Carbohydrate | Macronutrients | 0.98 (0.94, 1.03) | 0.512 |
| Englyst fibre | Macronutrients | 0.95 (0.90, 0.99) | 0.019 |
| Starch | Macronutrients | 0.96 (0.91, 1.00) | 0.060 |
| Total fat | Macronutrients | 0.97 (0.92, 1.01) | 0.150 |
| Total nitrogen | Macronutrients | 0.96 (0.92, 1.01) | 0.124 |
| Total protein | Macronutrients | 0.97 (0.92, 1.01) | 0.140 |
| Vegetable fat | Macronutrients | 0.93 (0.89, 0.98) | 0.003 |
| Vegetable protein | Macronutrients | 0.93 (0.89, 0.97) | 0.002 |
| Calcium | Minerals | 0.99 (0.94, 1.04) | 0.662 |
| Chloride | Minerals | 0.97 (0.93, 1.02) | 0.212 |
| Copper | Minerals | 0.94 (0.90, 0.98) | 0.010 |
| Nutrients | Group | *HR* (95% *CI*) | *P*-value |
| Haem iron | Minerals | 1.02 (0.97, 1.07) | 0.367 |
| Iodine | Minerals | 0.96 (0.92, 1.01) | 0.086 |
| Iron | Minerals | 0.93 (0.89, 0.98) | 0.003 |
| Magnesium | Minerals | 0.92 (0.88, 0.97) | 0.001 |
| Manganese | Minerals | 0.93 (0.89, 0.98) | 0.003 |
| Non-haem iron | Minerals | 0.93 (0.89, 0.97) | 0.002 |
| Phosphorus | Minerals | 0.97 (0.93, 1.02) | 0.237 |
| Potassium | Minerals | 0.95 (0.91, 1.00) | 0.049 |
| Selenium | Minerals | 0.91 (0.87, 0.96) | <0.001 |
| Sodium | Minerals | 0.98 (0.94, 1.03) | 0.505 |
| Zinc | Minerals | 0.99 (0.95, 1.04) | 0.714 |
| Alcohol | Others | 0.89 (0.85, 0.94) | <0.001 |
| Cholesterol | Others | 1.00 (0.95, 1.04) | 0.891 |
| Total weight of all foods and beverages | Others | 1.05 (1.00, 1.10) | 0.045 |
| Total weight of beverages only | Others | 1.09 (1.04, 1.14) | 0.001 |
| Free sugar | Sugars | 1.01 (0.97, 1.06) | 0.620 |
| Fructose | Sugars | 0.97 (0.93, 1.02) | 0.218 |
| Glucose | Sugars | 0.97 (0.93, 1.02) | 0.286 |
| Intrinsic and milk sugars | Sugars | 1.00 (0.95, 1.04) | 0.856 |
| Lactose | Sugars | 1.05 (1.00, 1.10) | 0.064 |
| Maltose | Sugars | 0.97 (0.92, 1.02) | 0.218 |
| Non-milk extrinsic sugars | Sugars | 1.01 (0.96, 1.06) | 0.763 |
| Other sugars | Sugars | 0.98 (0.94, 1.03) | 0.484 |
| Sucrose | Sugars | 1.03 (0.98, 1.07) | 0.306 |
| Total sugars | Sugars | 1.01 (0.96, 1.05) | 0.800 |
| Biotin | Vitamins | 0.93 (0.88, 0.97) | 0.002 |
| Nutrients | Group | *HR* (95% *CI*) | *P*-value |
| Folate | Vitamins | 0.96 (0.92, 1.01) | 0.131 |
| Niacin equivalent | Vitamins | 0.95 (0.91, 1.00) | 0.042 |
| Pantothenic acid | Vitamins | 1.01 (0.97, 1.06) | 0.603 |
| Retinol | Vitamins | 0.99 (0.95, 1.04) | 0.783 |
| Riboflavin | Vitamins | 1.02 (0.98, 1.07) | 0.333 |
| Thiamin | Vitamins | 0.98 (0.94, 1.03) | 0.477 |
| Vitamin A retinol equivalents | Vitamins | 1.02 (0.97, 1.07) | 0.477 |
| Vitamin B12 | Vitamins | 0.98 (0.93, 1.03) | 0.393 |
| Vitamin B6 | Vitamins | 1.00 (0.95, 1.05) | 0.943 |
| Vitamin C | Vitamins | 0.95 (0.91, 1.00) | 0.035 |
| Vitamin D | Vitamins | 0.95 (0.91, 1.00) | 0.033 |
| Vitamin E | Vitamins | 0.94 (0.90, 0.98) | 0.008 |

Models were adjusted for age, sex, ethnicity, education, TDI, smoking status, physical activity, sleep duration, hypertension, diabetes, and hyperlipidemia.

Abbreviations: CI, confidence interval; HR, hazard ratio.

**Supplementary Table 7. The associations between NRS and risk of RA after excluding participants with missing data (N = 130,562)**

| NRS | Cases/N | Model 1 | |  | Model 2 | |  | Model 3 | |
| --- | --- | --- | --- | --- | --- | --- | --- | --- | --- |
|  |  | HR (95% CI) | *P*-value |  | HR (95% CI) | *P*-value |  | HR (95% CI) | *P*-value |
| Continuous | 1,310/130,562 | 1.92 (1.56, 2.35) | <0.001 |  | 1.83 (1.49, 2.25) | <0.001 |  | 1.77 (1.44, 2.18) | <0.001 |
| Quartile |  |  |  |  |  |  |  |  |  |
| Q1 | 246/32,641 | 1 (Reference) |  |  | 1 (Reference) |  |  | 1 (Reference) |  |
| Q2 | 264/32,640 | 0.96 (0.81, 1.15) | 0.674 |  | 0.97 (0.82, 1.16) | 0.761 |  | 0.97 (0.81, 1.15) | 0.728 |
| Q3 | 362/32,640 | 1.25 (1.06, 1.47) | 0.007 |  | 1.25 (1.06, 1.48) | 0.008 |  | 1.23 (1.05, 1.45) | 0.013 |
| Q4 | 438/32,641 | 1.47 (1.25, 1.72) | <0.001 |  | 1.44 (1.23, 1.69) | <0.001 |  | 1.41 (1.20, 1.66) | <0.001 |
| *P* for trend |  |  | <0.001 |  |  | <0.001 |  |  | <0.001 |

Model 1 adjusted for age, sex.

Model 2 adjusted model 1 + ethnicity, education, TDI, smoking status, physical activity, and sleep duration.

Model 3 adjusted model 2 + hypertension, diabetes, and hyperlipidemia.

Abbreviations: CI, confidence interval; HR, hazard ratio; Q, quartile.

**Supplementary Table 8. The associations between NRS and risk of RA after excluding participants who experienced a RA within the first two years of follow-up (N = 161,592)**

| NRS | Cases/N | Model 1 | |  | Model 2 | |  | Model 3 | |
| --- | --- | --- | --- | --- | --- | --- | --- | --- | --- |
|  |  | HR (95% CI) | *P*-value |  | HR (95% CI) | *P*-value |  | HR (95% CI) | *P*-value |
| Continuous | 1,623/161,592 | 2.15 (1.79, 2.58) | <0.001 |  | 2.00 (1.66, 2.40) | <0.001 |  | 1.94 (1.61, 2.33) | <0.001 |
| Quartile |  |  |  |  |  |  |  |  |  |
| Q1 | 290/40,398 | 1 (Reference) |  |  | 1 (Reference) |  |  | 1 (Reference) |  |
| Q2 | 346/40,398 | 1.07 (0.92, 1.26) | 0.371 |  | 1.08 (0.92, 1.26) | 0.344 |  | 1.07 (0.92, 1.26) | 0.373 |
| Q3 | 424/40,398 | 1.25 (1.08, 1.46) | 0.004 |  | 1.24 (1.07, 1.45) | 0.005 |  | 1.22 (1.05, 1.43) | 0.009 |
| Q4 | 563/40,398 | 1.62 (1.40, 1.87) | <0.001 |  | 1.56 (1.35, 1.80) | <0.001 |  | 1.52 (1.32, 1.77) | <0.001 |
| *P* for trend |  |  | <0.001 |  |  | <0.001 |  |  | <0.001 |

Model 1 adjusted for age, sex.

Model 2 adjusted model 1 + ethnicity, education, TDI, smoking status, physical activity, and sleep duration.

Model 3 adjusted model 2 + hypertension, diabetes, and hyperlipidemia.

Abbreviations: CI, confidence interval; HR, hazard ratio; Q, quartile.

**Supplementary Table 9. Association between NRS and risk of RA using competitive risk model (N = 161,725)**

| NRS | Cases/N | Model 1 | |  | Model 2 | |  | Model 3 | |
| --- | --- | --- | --- | --- | --- | --- | --- | --- | --- |
|  |  | HR (95% CI) | *P*-value |  | HR (95% CI) | *P*-value |  | HR (95% CI) | *P*-value |
| Continuous | 1,756/161,725 | 2.12 (1.77, 2.53) | <0.001 |  | 1.98 (1.66, 2.36) | <0.001 |  | 1.92 (1.61, 2.29) | <0.001 |
| Quartile |  |  |  |  |  |  |  |  |  |
| Q1 | 314/40,432 | 1 (Reference) |  |  | 1 (Reference) |  |  | 1 (Reference) |  |
| Q2 | 368/40,431 | 1.05 (0.90, 1.22) | 0.011 |  | 1.05 (0.91, 1.23) | 0.490 |  | 1.05 (0.90, 1.22) | 0.530 |
| Q3 | 460/40,431 | 1.24 (1.08, 1.44) | <0.001 |  | 1.24 (1.07, 1.43) | 0.004 |  | 1.22 (1.05, 1.41) | 0.008 |
| Q4 | 614/40,431 | 1.61 (1.40, 1.85) | <0.001 |  | 1.55 (1.35, 1.78) | <0.001 |  | 1.52 (1.32, 1.74) | <0.001 |
| *P* for trend |  |  | <0.001 |  |  | <0.001 |  |  | <0.001 |

Model 1 adjusted for age, sex.

Model 2 adjusted model 1 + ethnicity, education, TDI, smoking status, physical activity, and sleep duration.

Model 3 adjusted model 2 + hypertension, diabetes, and hyperlipidemia.

Abbreviations: CI, confidence interval; HR, hazard ratio; Q, quartile.

**Supplementary Table 10. The associations between PRS and risk of RA**

| PRS | Model 1 | |  | Model 2 | |  | Model 3 | |
| --- | --- | --- | --- | --- | --- | --- | --- | --- |
|  | HR (95% CI) | *P*-value |  | HR (95% CI) | *P*-value |  | HR (95% CI) | *P*-value |
| Continuous | 1.34 (1.28, 1.41) | <0.001 |  | 1.35 (1.29, 1.41) | <0.001 |  | 1.34 (1.28, 1.41) | <0.001 |
| Quartile |  |  |  |  |  |  |  |  |
| Low | 1 (Reference) |  |  | 1 (Reference) |  |  | 1 (Reference) |  |
| Medium | 1.20 (1.06, 1.36) | 0.005 |  | 1.20 (1.06, 1.37) | 0.005 |  | 1.20 (1.06, 1.36) | 0.005 |
| High | 1.88 (1.67, 2.11) | <0.001 |  | 1.88 (1.67, 2.11) | <0.001 |  | 1.87 (1.67, 2.10) | <0.001 |
| *P* for trend |  | <0.001 |  |  | <0.001 |  |  | <0.001 |

Model 1 adjusted for age, sex.

Model 2 adjusted model 1 + ethnicity, education, TDI, smoking status, physical activity, and sleep duration.

Model 3 adjusted model 2 + hypertension, diabetes, and hyperlipidemia.

Abbreviations: CI, confidence interval; HR, hazard ratio; Q, quartile.

**Supplementary Table 11. Additive interaction between NRS and PRS on the RA risk**

| NRS & genetic risk | RERI (95% CI) | AP (95% CI) | SI (95% CI) |
| --- | --- | --- | --- |
| **Medium** **genetic risk** |  |  |  |
| Q2 | 0.12 (-0.41, 0.65) | 0.07 (-0.25, 0.40) | 1.23 (0.44, 3.44) |
| Q3 | 0.17 (-0.36, 0.70) | 0.09 (-0.20, 0.38) | 1.25 (0.56, 2.79) |
| Q4 | -0.25 (-0.87, 0.36) | -0.12 (-0.40, 0.16) | 0.82 (0.54, 1.26) |
| **High genetic risk** |  |  |  |
| Q2 | -0.54 (-1.25, 0.17) | -0.23 (-0.52, 0.07) | 0.72 (0.50, 1.04) |
| Q3 | -0.36 (-1.06, 0.34) | -0.13 (-0.38, 0.12) | 0.83 (0.60, 1.15) |
| Q4 | -0.54 (-1.30, 0.22) | -0.16 (-0.38, 0.06) | 0.81 (0.63, 1.06) |

Models were adjusted for age, sex, ethnicity, education, TDI, smoking status, physical activity, sleep duration, hypertension, diabetes, and hyperlipidemia.

Abbreviations: RERI, relative excess risk due to interaction; AP, attributable proportion due to interaction; SI, synergy index.

**Supplementary Table 12. Multiplicative interaction between NRS and PRS on the RA risk**

| Variable | HR (95% CI) | *P*-value | *P-*interaction |
| --- | --- | --- | --- |
| NRS | 3.14 (2.23, 4.42) | <0.001 |  |
| PRS |  |  |  |
| Low | 1 (Reference) |  |  |
| Medium | 1.25 (1.10, 1.44) | 0.001 |  |
| High | 1.98 (1.75, 2.24) | <0.001 |  |
| NRS × PRS |  |  | 0.005 |
| NRS × PRS (Medium vs Low) | 0.60 (0.37, 0.95) | 0.028 |  |
| NRS × PRS (High vs Low) | 0.49 (0.32, 0.75) | 0.001 |  |

Models were adjusted for age, sex, ethnicity, education, TDI, smoking status, physical activity, sleep duration, hypertension, diabetes, and hyperlipidemia.
